# CHANGE IN PHYSICAL FUNCTION, SWALLOWING, AND NUTRITIONAL RISK IN PEOPLE WITH PARKINSON’S DISEASE OVER 4 YEARS

**DOI:** 10.1093/geroni/igz038.3398

**Published:** 2019-11-08

**Authors:** Dara L LoBuono, Sarah Sarah Dobiszewski, Alison Tovar, Skye N Leedahl, Matthew J Delmonico, Furong Xu, Mahler Leslie, Ingrid E Lofgren

**Affiliations:** University of Rhode Island, Kingston, Rhode Island, United States

## Abstract

Parkinson’s disease, a neurodegenerative disease, impacts physical function, swallowing, and nutritional risk, but research has not examined how these health markers change longitudinally in one study. This study was an observational, longitudinal study with assessments conducted every 6 months over 4 years. The assessments included: the short physical performance battery (SPPB) (scores 75)), and weight in pounds. A one-way repeated measures ANOVA compared outcomes variables at baseline, years 2 and 4. Partial eta square are reported for effect size. Eight participants, baseline mean age 67.1±4.0 years and time since diagnosis=8.1±7.5 years, were assessed. At baseline, 3 participants exhibited possible mobility issues (SPPB=9.9±1.8), 2 participants exhibited a possible swallow issue (swallow speed=15.7±9.8mL/s), and 7 were at possible or at nutrition risk (64.0±13.8). At year 4, 5 participants exhibited possible mobility issues (SPPB=8.1±3.2, (ηp2=0.5), 4 participants exhibited a possible swallow issue (swallow speed=13.4±10.4 ml/s, ηp2=0.3), and there was a decline in perceived swallow function (77.3±12.4 vs. 74.8±16.2, ηp2=0.5). Six participants were at possible or at nutrition risk. Participants experienced weight declines from baseline to year 4 (178.5±31.4 vs.168.8±24.9, ηp2=0.5). The demonstrated decline in physical and swallow functioning, presence of nutrition risk, and experienced weight loss suggests that interdisciplinary intervention strategies may need to be designed and tested in this population.

